# Porcelain Fillings

**Published:** 1896-11

**Authors:** Elof Förberg

**Affiliations:** Stockholm, Sweden


### PORCELAIN FILLINGS.

BY ELOF FÖRBERG, STOCKHOLM, SWEDEN.

I HAVE been using porcelain fillings on a large scale since 1883. The material I use is of three kinds: (1) artificial teeth; (2) cylindrical bars; (3) body and enamel for fusing. Of artificial teeth the English ones, I consider, are the best, as their substance is more homogeneous than that of the American, and they are therefore more suitable for grinding. I use the American (How's) crown only when it is a question of repairing a broken corner of an incisor, where more or less of the cutting edge has to be replaced, and when, therefore, with a view to strength, platinum pins are considered requisite. This crown is hollowed out at the back, and therefore very thin, and it is provided with four thin, pliable platinum pins, which can advantageously be used as fastenings for the porcelain filling.

My method of using parts of artificial teeth as fillings has been explained by me at the meeting of the American Dental Society of Europe, at Coblenz, in 1887. It is briefly as follows: Having completely excavated the cavity and finished the edges, I take a piece of colored paper, or somewhat thick tin-foil, and hold this over the tooth surface with one hand, while with the other hand I rub the paper with a polishing-steel, so that the edges of the cavity cut through the paper. In this way I get a mould which perfectly shows the form of the cavity. This mould of paper or tin-foil is then stuck on, with varnish or something similar, to an artificial tooth, the color of which corresponds with that of the tooth to be fitted, when it should be observed that the curvature of the surface of the artificial tooth should be tolerably in accordance with that of the natural tooth; it is preferable, especially in case of a larger defect, to place the mould on that part of the artificial tooth which corresponds with the position of the defect of the natural tooth.

Going by the mould, glued onto the tooth, it will now be easy, by using cutting pliers and corundum or carborundum wheels, to form an inlay that will perfectly fit the cavity, and this can be done in the laboratory, so that all that remains to be done in the operating-room are the finishing touches, which must be from the cavity direct; before doing so the mould should naturally be removed, and the inlay fastened on a metal mandrel with gum-lac or a mixture of wax, rosin, and gutta-percha, which mixture is very suitable for sticking on pieces with (as well as for using as temporary fillings). When the piece has been accurately fitted, and everything has been made ready for the filling, the piece should be fastened in the cavity with Harvard cement. The cement, which should be well stirred and rather thin, is applied around the edges of the piece, which is then quickly pressed into the cavity, where it should be held with a steady pressure for some minutes to prevent its being displaced by compressed air or otherwise before the cement hardens. Only a thin layer of cement now separates the tooth from the porcelain inlay. The color of the cement should be a shade darker than that of the tooth. When How's crowns are used the piece should, of course, be fitted so that one or two of the platinum pins may be used as fastenings.

If, when, for instance, repairing a partly broken tooth, it is desired to have, on the palatine or lingual side, a material which, better than cement, can resist wear, the cement on that side may be covered with a thin layer of amalgam, or with gold, or one can, before inserting the piece, add melted glass or porcelain, thus forming the contour of the tooth. This course of action is preferable to making the parts entirely of glass or porcelain, inasmuch as one then gets a harder and stronger piece, and it is also then easier to get precisely the shade of color that is wanted.

The porcelain cylinders are naturally intended for cavities which have or can be given a cylindrical shape: thus, particularly for incisors with enamel defects, etc. The cylinders should be about twenty millimetres long and from one and a half to four millimetres in diameter, or nearly corresponding with the inlay drills that are met with in the market. The porcelain substance ought to be thoroughly burned, so that on breaking or grinding it one gets a firm and bright surface, and should be of such shades of color as are mostly met with. My method of using such porcelain cylinders, for which method I claim priority, is as follows: A cylinder, of suitable color and a little thicker than the drill used, is fixed in a porte-polisher with gum-lac or the gutta-percha mixture previously referred to. When rotating in the machine the cylinder is clasped round with a corundum cloth held by the left hand, and is thus ground (slightly conical) until it can just be pushed into the cavity. The cylinder is then moistened with water or glycerin, is dipped into fine carborundum powder, and, while rotating, is introduced into the cavity, where it will then be perfectly ground in. In doing this the hand-piece must be held very steadily, with only a slight pressure on the cavity to prevent the cylinder from breaking. The corundum powder is then washed away with water, the cavity is dried, and the cylinder tried. The end of the cylinder is now ground off somewhat, so that the piece, when placed, rests close against the walls of the cavity. It is better not to use too fine a corundum wheel for this grinding, as the cement will adhere better on a rough surface. If the cavity is not too shallow no other fastenings are required for the piece. The cylinder is then marked to the depth it enters into the cavity, and it is then cut nearly off at that place with a thin diamond or carborundum wheel. A thin layer of cement or gutta-percha solution is then applied round the sides of the cylinder, which is then inserted into the cavity with a firm pressure, and held there until the cement has partially hardened. With a slight movement of the hand the cylinder is then broken off at the place cut through. Some melted wax or paraffin is then poured on with a spoon-shaped instrument. The piece is not polished until the cement has hardened well. If these cylinder fillings are carefully done and well polished with Arkansas stone, etc., it is quite impossible to distinguish them from the natural tooth when standing a few paces off. The advantage of this method with cylinders instead of common inlays is that the cylinder can safely be fixed in a porte-polisher, and thus ground against a corundum wheel and cloth, as well as in the cavity itself, without the risk of slipping, which, with small inlays, generally is the case, even though they be fixed on metal mandrels. The cylinders can also be used for cavities of an oval or other shape when they are ground in as common inlays and cut off as previously described. Even then one has the advantage of being able to hold the piece well fast while working.

The way of using enamel for moulding is well known. I only want to mention a few details of my way of procedure. Having pressed the gold-platinum-foil well up, thereby using cotton and polishers, I pour some hard wax (consisting of one part of wax and two parts of rosin) into the mould thus obtained. I then pack the wax well, while it is hardening, and also press it over the surface of the tooth. By this means I get a very good cast of the cavity, and also prevent the gold-platinum mould from altering shape while being taken out of the cavity; and, lastly, I can get a plaster-cast model of the tooth, whereby I can better see the shape of it when forming and fusing the porcelain filling. I now mostly use Reisert's enamel, which is melted in an oven. In order to obtain a natural color a somewhat yellow enamel should be used at the bottom, and on top of that the color chosen. I generally also add some continuous-gum body to the first layer. I formerly used a heated instrument for pressing the inlay into the cavity, but it seemed to me as if that rendered the cement less durable. In cases of approximal cavities I generally use a thin linen band for pressing in the piece and removing the superfluous cement.

The question as to what cavities are particularly suitable for porcelain and glass fillings I think I must answer as follows: Fillings of the kinds mentioned are suitable for visible defects in the incisors, cuspids, and (possibly) bicuspids. I have used Roustaig's cement as long as it could be had, and afterwards Harvard cement. As regards the question how long a time these fillings will last, it depends upon three factors,—viz., (1) the condition of the cavity, (2) the degree of thinness to which one has succeeded in reducing the cement layer, and (3) the amount of attention the patient gives to his mouth.

The cavities should not be too shallow (especially not cervical cavities, in which it is, as a rule, difficult to get durable fillings). I have several cases where porcelain fillings, in larger cervical cavities of irregular shape as well as in cavities which cover the whole of the approximal surface and half of the cutting edge of incisors have lasted ten to twelve years, nothing having been done to them in the mean time excepting refreshing the cement stripe a little once or twice. The most durable are naturally the cylinder fillings, because, having rotated and ground itself into the cavity, it fits so perfectly that the cement layer is absolutely as fine as a hair. Here, as elsewhere, hygiene plays an important part. To insert a series of porcelain fillings into cervical cavities on a person who, on account of neglect (or of chronic gingivitis), has the cervical parts of his teeth covered with deposits is, I consider, labor totally thrown away.

The last question, whether porcelain fillings can be considered as a satisfactory substitute for gold, aside from æsthetic considerations, has previously been partly answered by the mentioning of porcelain fillings which are now ten to twelve years old, and which, by all appearances, may last as long again. It may generally be said that porcelain fillings can rival gold fillings in durability in cases where they, with a minimum cement layer, touch upon tolerably firm enamel edges, and where, in biting, their surface does not articulate against the opposite teeth.

